# Informed cover measurement: Guidelines and error for point‐intercept approaches

**DOI:** 10.1002/aps3.11446

**Published:** 2021-11-01

**Authors:** Taly Dawn Drezner, Zvi Drezner

**Affiliations:** ^1^ Department of Physical Sciences, College of Southern Nevada 6375 West Charleston Boulevard Las Vegas Nevada 89146 USA; ^2^ College of Business and Economics California State University‐Fullerton Fullerton California 92834 USA

**Keywords:** ecosystem modeling, field methods, measurement error, point‐intercept method, simulation modeling, species cover, statistical methods

## Abstract

**Premise:**

The point‐intercept method is one of the most commonly used approaches to measure species cover in ecosystems worldwide. In this approach, multiple points are sampled for presence/absence of a species, and the number of present points divided by the total number of sampled points provides an estimate of percent cover. Our purpose is to mathematically analyze the accuracy of the point‐intercept approach and establish guidelines for its use.

**Methods:**

We developed formulas that analyze the point‐intercept method and confirmed their effectiveness using simulations.

**Results:**

We find that a point‐intercept spacing of at least 80% of the largest plant diameter provides the most reliable results. We present a user‐friendly spreadsheet that calculates the number of intercepts needed for fieldwork, as well as the standard deviation, expected deviation, and confidence interval of the collected data.

**Discussion:**

We provide a variety of guidelines for establishing field protocols based on our results, including dealing with rare species and combining results for multiple species. Quadrat characteristics (intercept spacing, number of point intercepts) can now be easily calculated to guide research design prior to fieldwork; after fieldwork is complete, the accuracy of this technique can (and should) be reported in all future ecological studies in which it is used.

The point‐intercept method (Mueller‐Dombois and Ellenberg, 1974, and references therein) and its variants (e.g., Evans and Love, 1957) are commonly used by ecologists to estimate plant cover in a given area, including for use in local and national monitoring programs (Karl et al., 2017). Cover is defined here as the area over which a certain species or multiple species occur; looking down from above, a certain portion of the ground is covered by the vegetation. The point‐intercept method is a non‐destructive sampling method where a quadrat or grid is constructed with defined points for sampling, such as pins that protrude down from a frame, or a framed grid of string with cross‐stitch intersections, or other designs that identify multiple points for sampling. The quadrat is placed randomly in the study area and each point is sampled independently for all species below or above that point; bare ground is noted where there is no vegetation. The percentage of point intercepts covered by a species provides an estimate of its cover in that area. The point intercepts can be arranged in a rectangular quadrat, or in a linear transect‐like pattern, as is common for rangelands (e.g., Roberts et al., 2021) that are dominated by grasses, or in other geometric alignments. Various sources provide more information about the point‐intercept method and other factors for measuring the cover and abundance of vegetation (e.g., Evans and Love, 1957; Mueller‐Dombois and Ellenberg, 1974). This approach can also be used for some animal groups such as coral (Zvuloni and Belmaker, 2016). The point‐intercept method has been employed across nearly all terrestrial (e.g., Radtke and Bolstad, 2001; Jackson et al., 2006; Sankey et al., 2014; Kent et al., 2018) and marine (e.g., Russ et al., 2015; Zvuloni and Belmaker, 2016) environments, although in certain environments (e.g., dense forest overstories) other approaches may be favored. The point‐intercept approach can also be used to detect other types of data, such as biomass (Jonasson, 1988; Barkaoui et al., 2013). This approach and its variants are not practical for estimating the cover for very rare or trace species, however; for example, if a forb occurs once or twice in a large study area or even a target sampling area, the probability of an intercept falling on an individual is very low. Furthermore, if an intercept did capture it, it would be over‐represented in the database. We discuss rare species in this study.

Studies have attempted to gauge the sampling error associated with the point‐intercept technique by comparing it to different methodological approaches; for example, Dethier et al. (1993) compared random point quadrats with visual estimates, while Lam et al. (2006) compared point‐intercept transects with video‐based estimates from randomly sampled frames in videos. Other studies compared field intercept sampling with a computer‐based sampling of images in which quadrats are superimposed on digital images of the area (Hulvey et al., 2018). Etchberger and Krausman (1997) compared different methods to their “known” cover, which was based on counts of all individual plants and their calculated percentage occurrence. Booth et al. (2006) compared traditional and automated methods and compared their results with “known” values, which they described as being derived from pictures taken 2 m above the ground; however, Hulvey et al. (2018) observed that layers of vegetation are poorly sampled using photography as lower‐lying vegetation may be missed. Point‐intercept techniques have also been used in conjunction with or compared with remotely sensed data (e.g., Karl et al., 2017); however, we question whether estimates from different field techniques should even be compared. Estimates from a particular method should be compared to the true cover, but there is no method to unequivocally obtain “true” cover or perhaps to see which methods are better than others with large samples (Applestein et al., 2018). In the current study, we overcome this problem using simulated areas, which allows us to precisely calculate the cover of simulated plants and study sites. Our simulation creates the cover and calculates its cover exactly, and then simulates point‐intercept sampling to compare.

Studies using the point‐intercept method vary greatly in the number of point intercepts used (e.g., 36 points [6 × 6], 100 points [10 × 10], etc.), the quadrat size (1 × 1 m, 5 × 5 m, etc.), and the spacing between the point intercepts (Kent et al., 2018; Lévesque et al., 2018). The number of point intercepts used in a study is essentially a cost‐benefit analysis; more data are better but require greater time and resource investments, while under‐sampling may compromise the results and integrity of the study. Ideally, quadrat attributes will vary with the characteristics of the community and species. At the research design stage, decision‐making is often based on past practice rather than specific knowledge of optimal protocols, and uncertainty in decision‐making has also been reported (e.g., Barkaoui et al., 2013). In this paper, we quantify the accuracy of the point‐intercept methodology and provide guidelines to aid researchers in establishing their field protocols and research design (Box 1). We do not advocate or discourage the use of this or any other approach; rather, we provide the underlying math to inform a researcher about the accuracy they can expect to achieve and, based on these results, we provide guidelines for research design. It is up to the researcher to judge their field site and needs in deciding which field method to apply, including deciding whether they prefer to report standard deviation, confidence intervals, or expected error. Each study is unique, and researchers must determine for themselves which approach is best for their data, their study, and their objectives. Researchers must make thoughtful decisions about their desired expected error, confidence interval size, or other measure, and given that there is no standard, this will have to be established in each study. There are no wrong answers as long as these measures of accuracy are reported. We provide a supplemental Excel file (Appendix S1) that calculates the necessary parameters for the study. Specifically:

BOX 1 **Overview and highlights of the procedures developed**

**Table:** 

**Use and purpose** ✓ We develop the statistical foundation for point‐intercept methods to guide researchers before and after field collection. ✓ We neither advocate nor discourage the use of this or any other approach. ✓ The accuracy of past studies can also be calculated. **Basic assumptions** ✓ Two leaves or plants of the same species that cover an intercept are counted once. ✓ Each species is counted independently, with cover ranging from 0% to 100%. **Research design points** ✓ Intercepts must *always* be spaced by ≥80% of the diameter of the widest plant both within and between quadrats. ✓ Sampling and quadrat distribution must *always* be random. ✓ Each species does not require a unique quadrat; for example, a quadrat for the largest understory species can be used for all understory species. ✓ Sampling of intercepts can be in any form (points along transects, any‐shaped quadrat, etc.). ✓ Intercepts should be widely distributed across the study site. A quadrat with fewer intercepts sampled several times may provide a better estimate than a single large quadrat. ✓ Species with around 50% cover require the largest number of sampled intercepts for the same accuracy. ✓ Most cover measurement methods miss rare species. When detected, they are assigned trace (1% or other) values. Further quantification may be impractical for any field technique. **The supplemental spreadsheet (Appendix S1) calculates**: ✓ BEFORE fieldwork: the number of intercepts required to obtain a desired level of accuracy for a study. ✓ AFTER fieldwork: the standard deviations, expected deviations, and confidence intervals for the data, as well as calculations for combining multiple species (including the same species in multiple canopy layers).

## Before fieldwork

1. We determine how many point intercepts are required to achieve a target level of accuracy (based on standard deviation, expected deviation, or confidence interval size).
2. We provide point‐intercept spacing guidelines that provide the greatest accuracy (least possible inaccuracy) in measurements for any given number of point intercepts.

## After fieldwork

1. The standard deviations, expected deviations, and confidence intervals for the field data results can be calculated using a supplemental spreadsheet or given formulas.
2. We provide methods for combining species cover calculations for the total cover of multiple species and the overall accuracy with a supplemental spreadsheet or formulas.
3. We provide guidelines for rare species assessments and calculations.

## METHODS AND RESULTS

In this study, we identify cover as the percentage of an area covered by a single species. If multiple leaves of the same plant or two plants of the same species cover the same point, the cover is counted once. If two different species cover the same point, then it is counted twice, once for each species. If a researcher chooses to count the overstory and understory separately, for example, then one species can effectively become two “species” (one in each sampled canopy layer), and if the cover is aggregated it could exceed 100%. We first calculate the cover by one species and then calculate the combined cover. Combined cover can be of different species (e.g., forbs) or the same species in multiple canopy layers. These calculations are applicable for all definitions of cover as determined by a researcher, as long as the definition is applied consistently within the study.

In order to build our model, we first develop three measures of accuracy: (1) expected deviation (*d*_*e*_): the expected difference, as an absolute value, between the measured cover and the actual cover; (2) standard deviation (*σ*): the standard deviation of the difference between the actual cover and the measured cover; and (3) confidence interval $\left(c-\frac{h}{2},c+\frac{h}{2}\right)$ of the actual cover for the measured cover. For definitions see Table 1. Next, we show how to calculate these three measures of accuracy.

**Table 1:** Definitions of the symbols mentioned in the text

Symbol	Definition
*n*	Total number of intercepts in a sample (one quadrat or several quadrats in an area)
*k*	Number of intercepts covered by a sampled species
*k/n*	Measured cover (% of intercepts covered by a species in a sample)
*p*	Actual cover (true cover estimated by a researcher) expressed as 0–100% or 0.0–1.0
α	Confidence level for the confidence interval
γ	$=\frac{z_{\alpha /2}^{2}}{n}$ where *z* is the standardized normal
*h*	Size of the confidence interval
*c*	Center of the confidence interval
*h*_*i*_	Size of the confidence interval of a single species *i*
*d*	Deviation (absolute value of the difference) between actual and measured cover
*d*_*i*_	Expected deviation of a single species *i*
*d*_*e*_	Expected deviation between actual and measured cover
$\hat{\sigma }$	Standard deviation of the combined cover of several species
*r*_*ij*_	Correlation coefficient between the covers of species *i* and species *j*

We begin by establishing the expected deviation. By definition, the probability that a particular point is covered is the (unknown) actual cover value, *p*. Each point intercept is a yes/no result, which is by definition a binomial distribution as long as they are not correlated; point intercepts are not correlated as long as they are appropriately spaced. We will later report our 80% rule for spacing, which eliminates the issue of correlation. Thus, the probability that *k* out of *n* points are covered, assuming no correlation, follows a binomial distribution (Neter et al., 1993):

(1) $$P\left(k\right)=\frac{n!}{k!(n-k)!}p^{k}{\left(1-p\right)}^{k}$$

When *p*, the probability of cover, is very low, 1 – *p* is about 1 and the binomial distribution converges to a Poisson distribution. The uncorrelated binomial distribution covers all options because each measurement is yes or no. The expected difference in absolute value between $\frac{k}{n}$ and *p* (expected deviation $E\left(\left|\frac{k}{n}-p\right|\right)$ is the sum of the deviations $\left|\frac{k}{n}-p\right|$, each multiplied by its probability:

(2) $$E\left(\left|\frac{k}{n}-p\right|\right)=\sum_{k=0}^{n}\left|\frac{k}{n}-p\right|P(k)$$

**Figure 1:**
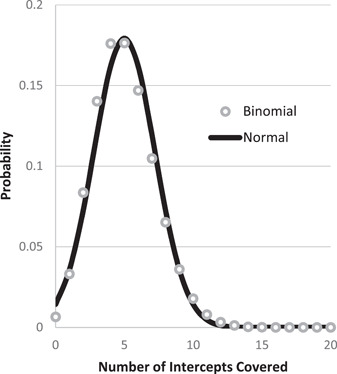
Comparing the binomial distribution for *n* = 500 intercepts (*p* = 1%) to a normal distribution

The confidence interval and expected error do not have a closed‐form expression when using the binomial distribution. Consequently, the required *n* cannot be estimated to achieve a given confidence interval or expected error when using the binomial distribution. We therefore apply a good approximation for *P(k)* (Equation (1)), which is obtained using the normal distribution (Figure 1). Such an approximation is more accurate when the expected number of covered points or non‐covered points is at least five (*np* > 5 and *n*(1 – *p*) > 5). The simulation was based on the binomial distribution, and its results confirmed the accuracy of the normal approximation.

Next, we calculate the expected deviation, *d*_*e*_. The proportion of covered points $\frac{k}{n}$ has a mean *p* with a standard deviation of $\sigma =\sqrt{\frac{p(1-p)}{n}}$. Using the normal approximation, the expected deviation is:

(3) $$d_{e}=E\left(\left|\frac{k}{n}-p\right|\right)=\frac{1}{\sqrt{2\pi }\sigma }\int_{-\infty }^{\infty }|x-p|e^{-\frac{{\left(x-p\right)}^{2}}{2\sigma^{2}}}\mathrm{dx}=\frac{2\sigma }{\sqrt{2\pi }}\int_{0}^{\infty }ze^{-\frac{z^{2}}{2}}\;\mathrm{dz}=\sqrt{\frac{2p(1-p)}{\pi n}}=\sqrt{\frac{2}{\pi }}\;\sigma .$$

In Equation (3), the expected deviation and the standard deviation satisfy $d_{e}=\sqrt{\frac{2}{\pi }}\;\sigma$; therefore, we only detail the calculation of *d*_*e*_, as *σ* is then calculated from this result. Appendix S1 calculates both the standard deviation and the expected deviation.

Next, we establish the formula for the confidence interval. The confidence interval for the estimated cover $\frac{k}{n}$ for a given (but unknown) actual cover, *p*, with a confidence level of 1 – α is (Neter et al., 1993):

(4) $$\hat{p}\pm z_{\alpha /2}\sqrt{\frac{\hat{p}(1-\hat{p})}{n}}$$

where $z_{\alpha /2}$ is the standardized normal distribution (e.g., *z* = 1.96 at a 95% confidence level).

Because *p* is unknown, the upper and lower limits of the confidence interval are denoted by $\hat{p}$ for a given $\frac{k}{n}$. By Equation (4), the limits of the confidence interval, denoted by $\hat{p}$, satisfy

$$\hat{p}\pm z_{\alpha /2}\sqrt{\frac{\hat{p}(1-\hat{p})}{n}}=\frac{k}{n}$$

leading to

$${\left(\hat{p}-\frac{k}{n}\right)}^{2}=\frac{z_{\alpha /2}^{2}}{n}\hat{p}(1-\hat{p})$$

Defining $\gamma =\frac{z_{\alpha /2}^{2}}{n}$, we get: $\left(1+\gamma \right){\hat{p}}^{2}-\left(2\frac{k}{n}+\gamma \right)\hat{p}+\frac{k^{2}}{n^{2}}=0$ whose solutions are:

(5) $$\hat{p}=\frac{2\frac{k}{n}+\gamma \pm \sqrt{{\left(2\frac{k}{n}+\gamma \right)}^{2}-4\frac{k^{2}}{n^{2}}(1+\gamma )}}{2+2\gamma }=\frac{2\frac{k}{n}+\gamma \pm \sqrt{4{\frac{k}{n}\gamma +\gamma }^{2}-4\frac{k^{2}}{n^{2}}\gamma }}{2+2\gamma }=\frac{2\frac{k}{n}+\gamma \pm \sqrt{\gamma }\sqrt{4\frac{k}{n}+\gamma -4\frac{k^{2}}{n^{2}}}}{2+2\gamma }=\frac{\frac{k}{n}+\frac{1}{2}\gamma }{1+\gamma }\pm \frac{\frac{1}{2}\sqrt{\gamma }}{1+\gamma }\sqrt{4\frac{k}{n}\left(1-\frac{k}{n}\right)+\gamma }=c\pm \frac{1}{2}h$$

where *c* is the center of the confidence interval and *h* is its size.

### The spacing of point intercepts

If one point intercept covers a plant, the probability that an adjacent point intercept covers the same plant increases when the point intercepts are spaced too closely together, resulting in a positive correlation between adjacent point intercepts. The number of covered point intercepts follows a binomial distribution when point intercepts are independent (Neter et al., 1993), but when they are positively correlated the variance of the correlated binomial distribution increases (Drezner, 2006; see Lemma 5 in Drezner and Farnum, 1993). That is, the accuracy of the cover estimate deteriorates when the point‐intercept spacing is inappropriately small for the plants being sampled. We calculate and establish appropriate spacing for point intercepts.

We begin by establishing the measures of accuracy for our simulation. Accuracy is measured by the expected deviation (*d*_*e*_) between the actual cover and the measured cover; for example, for an actual cover of 10%, a measured cover of 11% or 9% would have a measured deviation of 1%. We also determine the best point‐intercept spacing through simulations of point‐intercept measurements for the following conditions: (i) actual cover of 10%, 20%, 30%,…, 90% (nine scenarios); (ii) point‐intercept spacing that is 10%, 20%, 30%,…, 200% of the diameter of the widest plant (20 scenarios) expected in a given study area; and (iii) four different numbers of point‐intercept points (25, 100, 225, or 400). These represent 720 (9 × 20 × 4) combinations.

An important element of our simulation and, we suggest, of real‐world data collection, is the necessity of randomly collected data. The distribution of a species does not affect the estimate of the sampled cover as long as the quadrats are placed randomly. By randomly, we refer to the absence of any bias on point selection and quadrat placement, ideally including replicates of a single quadrat. For example, ten 20‐point quadrats are better than one 200‐point quadrat. Not selecting the quadrats randomly is referred to as biased sampling. If a species has a non‐random or clumped distribution, this issue would not affect the cover estimate so long as the sampled points were selected randomly. The point‐intercept method captures cover accurately, even if a species has a clumped or other distribution, as long as the sampling is random. If points are distributed over a sub‐area (e.g., a woody patch) of the study site, then estimates will be valid for that sub‐area. Researchers should consider using a quadrat with a smaller number of point intercepts and sampling over the whole study area, maintaining minimum spacing rules (see Goslee [2006] for a discussion on clumping).

We begin by simulating a region with randomly generated plant diameters to create plants of different sizes. Individual diameters were established around a mean (the diameters ranged from 50% to 150% of the mean) for that species. A maximum canopy width of the largest individual was established for the simulated species, and its size range across individuals was set to 33% to 100% of its maximum determined width. Plants of varying sizes were generated in random locations until the total cover in that area had one of the given cover values (e.g., 10%). This produced about 1000–20,000 plants (higher numbers for higher cover values) in the simulated region. We simulate 100 of these unique regions for each of the 720 combinations, and then randomly sample 1000 quadrats inside each region, thus sampling 100,000 quadrats for each of the 720 combinations, for a total of 72 million randomly generated quadrats. Two cover values are obtained with this approach: (1) estimated cover for each quadrat, which is found by point‐intercept sampling and represents measured cover, and (2) the actual (real) cover of the region, which can be calculated exactly as it is generated by the simulation. A FORTRAN program that performs the simulation is provided in Appendix 1, and the simulation details are described in Appendix 2.

**Figure 2:**
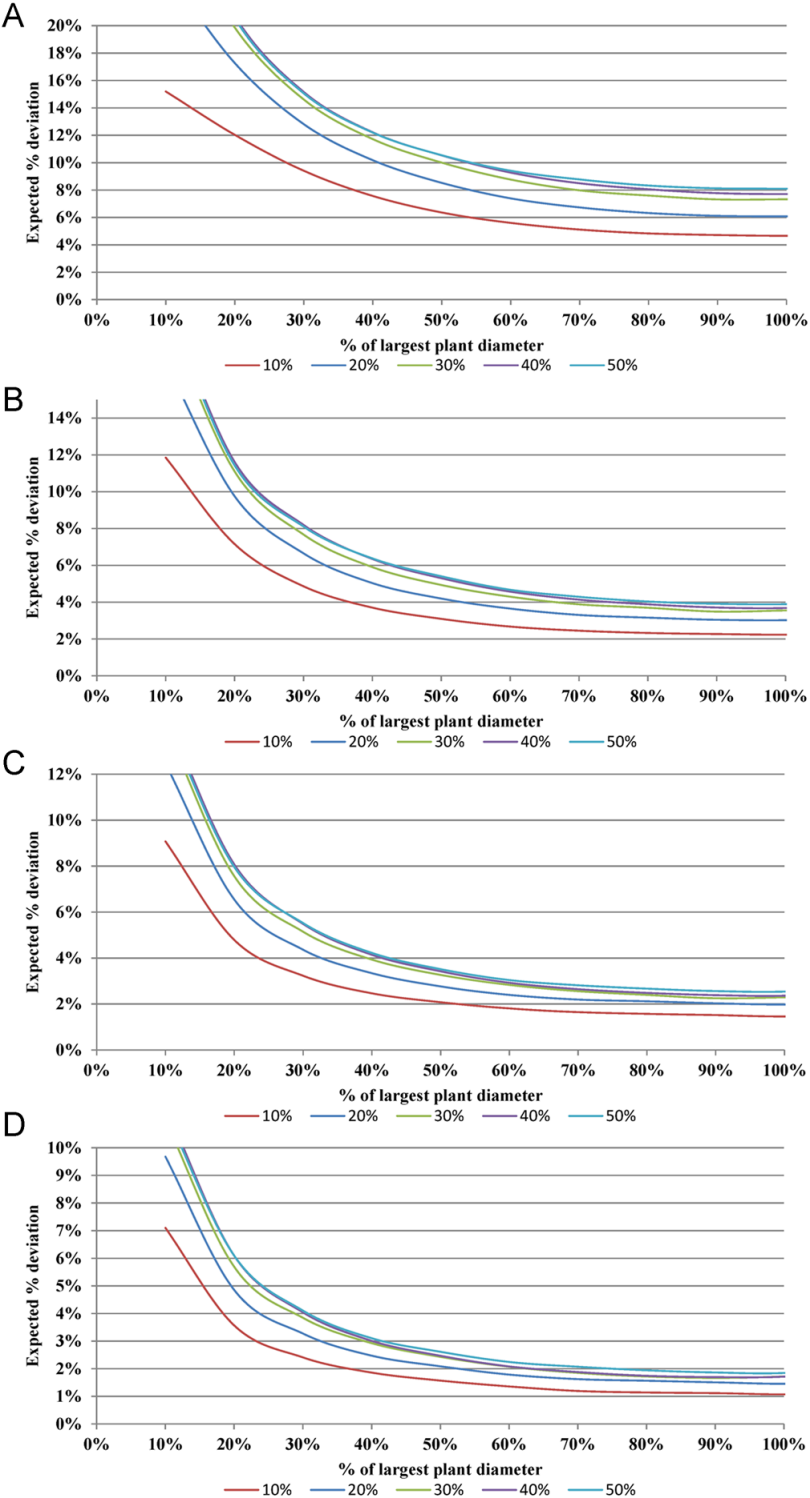
Simulated expected deviations (%) of the measured cover from the actual cover for actual covers ranging from 10% (lowest line) to 50% (highest line) in 10% increments. The 40% and 50% lines are very close together, and thus appear as a thicker line. (A–D) Values for (A) 25, (B) 100, (C) 225, and (D) 400 intercepts. Values up to 50% cover are given, as the values for complements are the same (e.g., the 80% line is the same as its complement, the 20% line). The lines continue beyond 100% of the largest plant diameter and are stable, and are thus truncated here to show more detail for the lower values

The expected deviation found by the simulation of nine cover values (10–90%) for each of the four tested numbers of point intercepts (25, 100, 225, and 400) are shown in Figure 2. The expected deviations stabilize to a horizontal line at a point‐intercept spacing greater than about 80% of the diameter of the widest plant in all 36 of these combinations. This takes into account the 20 point‐intercept spacing values, and therefore indicates that the optimal spacing should be at or above 80% of the diameter of the widest plant. The accuracy of the field data deteriorates below 80% spacing (Figure 2), but is stable above 80%. In the remainder of this paper, we assume that the point‐intercept spacing is at least 80% of the maximum plant diameter.

The standard deviation also stabilizes at about 80% of the widest plant, as seen through Equation (3), where the standard deviation of a binomial distribution is $\sqrt{\frac{p(1-p)}{n}}=\sqrt{\frac{\pi }{2}}d_{e}$. The exact values for Equation (2) compared with the approximation values (Equation (3)) of *p* = 10%, 20%, …, 90%, and many values of *n* ≥ 25 confirms the accuracy of the normal approximation. The simulated results in Table 2 also confirm the accuracy of Equation (3) when the point‐intercept spacing is greater than 80%. The values calculated using Equation (3) match the values of the horizontal lines in Figure 2.

**Table 2:** Simulation results of the average deviation of the measured cover and actual cover for intercept spacing between 100% and 200% of the largest plant diameter compared with Equation (3). The examples below are for 25 (e.g., 5 × 5), 100 (e.g., 10 × 10), 225 (e.g., 15 × 15), and 400 (e.g., 20 × 20) intercepts (*n*)

	*n* = 25		*n* = 100		*n* = 225		*n* = 400	
Cover percentage	Simulation result	Equation (3) result	Simulation result	Equation (3) result	Simulation result	Equation (3) result	Simulation result	Equation (3) result
10%	4.8%	4.8%	2.3%	2.4%	1.5%	1.6%	1.1%	1.2%
20%	6.2%	6.4%	3.1%	3.2%	2.1%	2.1%	1.6%	1.6%
30%	7.4%	7.3%	3.6%	3.7%	2.4%	2.4%	1.8%	1.8%
40%	7.7%	7.8%	3.8%	3.9%	2.5%	2.6%	1.8%	2.0%
50%	8.1%	8.0%	3.9%	4.0%	2.6%	2.7%	1.9%	2.0%

### Establishing the number of point intercepts for fieldwork

To establish field protocols, measurement error can be expressed in three ways: (1) the expected deviation (absolute deviation of the measured cover from the actual cover), (2) standard deviation, and (3) the confidence interval of the actual cover for a given measured cover. The researcher chooses their target expected deviation, standard deviation, or confidence interval size for their final field measurement statistics, and the supplemental Excel file (or Equations ((6), (7), (9), and 10)) is used to calculate the number of point intercepts required to achieve that target.

#### Expected deviation and standard deviation

Equation (3) calculates the expected deviation (*d*_*e*_) for any given actual cover 0 ≤ *p* ≤ 1 (0–100%) of a species. To find the number of point intercepts (*n*) required to obtain a certain value of *d*_*e*_, we solve Equation (3) for *n* and get:

(6) $$n=\frac{2p(1-p)}{\pi d_{e}^{2}}$$

The largest value of *n* is obtained for *p* = 0.50 (for which $\left(1-p\right)=\frac{1}{4}$); therefore, for any *p*,

(7) $$n\leq \frac{2\times \frac{1}{4}}{\pi d_{e}^{2}}=\frac{1}{2\pi d_{e}^{2}}$$

Equation (7) uses *p* = 0.50, making it the most conservative estimate of the number of point intercepts needed, and is thus valid for any cover value. If a researcher is confident that a species has well above or below 50% cover, fewer point intercepts are needed to achieve a given *d*_*e*_, and Equation (6) can be used. The values of *n* for Equations (6) and (7) are both calculated in Appendix S1.

In Equation (3), the standard deviation is $\sigma =\sqrt{\frac{\pi }{2}}d_{e}$; thus, the number of point intercepts required by a target standard deviation *σ* is $\frac{4}{\pi }$ of the values calculated using Equations (6) and (7) with the expected deviation (*d*_*e*_). The number of point intercepts required for a target standard deviation is also calculated in Appendix S1.

#### Confidence interval size

We establish the formula that a researcher can use to calculate the number of point intercepts, *n*, required to achieve a confidence interval size *h* (Table 1). Based on Equation (5):

(8) $$h=\frac{\sqrt{\gamma }}{1+\gamma }\sqrt{4\frac{k}{n}\left(1-\frac{k}{n}\right)+\gamma }$$

Squaring this equation yields a quadratic equation in *γ*. For a relatively large *n*, $\gamma =\frac{z_{\alpha /2}^{2}}{n}$ is small and thus $\frac{\sqrt{4\frac{k}{n}\left(1-\frac{k}{n}\right)+\gamma }}{1+\gamma }\;\;\approx \;$ $\sqrt{4\frac{k}{n}\left(1-\frac{k}{n}\right)}$, leading to a much simpler expression:

(9) $$\sqrt{\gamma }\sqrt{4\frac{k}{n}\left(1-\frac{k}{n}\right)}=h\to \gamma =\frac{z_{\alpha /2}^{2}}{n}\approx \frac{h^{2}}{4\frac{k}{n}\left(1-\frac{k}{n}\right)}\to n\approx \frac{4\frac{k}{n}\left(1-\frac{k}{n}\right)z_{\alpha /2}^{2}}{h^{2}}$$

Because $\frac{k}{n}\left(1-\frac{k}{n}\right)\leq \frac{1}{4}$ for any $\frac{k}{n}$, substituting $\frac{1}{4}$ for $\frac{k}{n}\left(1-\frac{k}{n}\right)$ in Equation (9) yields the conservative estimate:

(10) $$n\leq \frac{z_{\alpha /2}^{2}}{h^{2}}$$

Like for the expected deviation, the most conservative value of *n* is obtained for $\frac{k}{n}=0.50$. The supplemental Excel file provided as Appendix S1 (calculated based on Equation (10)) can thus be used to calculate *n* for any measured cover. However, if the researcher is confident that the measured cover, $\frac{k}{n}$, of a species will not exceed a specific value below 50%, or will exceed a specific value above 50%, the supplemental spreadsheet based on Equation (9) should be used.

### Accuracy of field data after data collection

The standard deviation (*σ*), expected deviation (*d*_*e*_), and the size of the confidence interval (*h*) are measures of accuracy that can be used to establish measurement error.

#### Expected deviation and standard deviation of collected data

To calculate the expected deviation (*d*_*e*_), we calculate the probability distribution of *k* (the number of point intercepts covered by a species) out of the total number of point intercepts sampled (*n*) in one or more quadrats, for an actual cover of 0 ≤ *p* ≤ 1 (0–100%). The ratio $\frac{k}{n}$ represents the field data and is a researcher's estimate of *p*. The expected deviation of $d_{e}=\left|\frac{k}{n}-p\right|$ is given in Equation (3). After data collection, we substitute the measured cover ($\frac{k}{n}$) for *p* in Equation (3). The standard deviation is $\sqrt{\frac{\pi }{2}}$ of the expected deviation.

#### Confidence interval of the actual cover

Equation (5) gives the confidence interval for the actual cover (*p*) at the confidence level 1 – α. The point estimate is $\frac{k}{n}$; however, the confidence interval is not symmetric around $\frac{k}{n}$, unless $\frac{k}{n}=0.50$. The center of the confidence interval is at distance $\frac{\gamma }{1+\gamma }|\frac{1}{2}-\frac{k}{n}|$ from the point estimate $\frac{k}{n}$.

**Figure 3:**
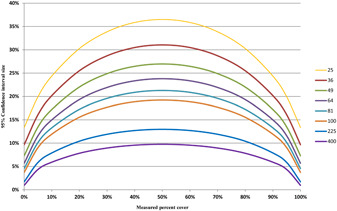
The size (*h*) of the 95% confidence interval for the selected values of *n* and all possible measured cover values calculated using Equation (5)

The sizes of the 95% confidence interval as a function of the measured cover for various values of *n* are shown in Figure 3. The size of the confidence interval is approximately inversely proportional to $\sqrt{n}$.

#### Combining multiple species

When several species are measured in an area (or one species is sampled independently in multiple canopies), the combined cover and its accuracy can be calculated. The number of species sampled in an area is defined as *t*. Species *i* (for *i* = 1,…, *t*) has a measured cover *p*_*i*_, with an expected deviation *d*_*i*_.

The mean of the combined cover is the sum of the individual covers with an expected deviation ${\hat{d}}_{e}$:

(11) $${\hat{d}}_{e}=\sqrt{\sum_{i=1}^{t}d_{i}^{2}}$$

The combined cover and its expected deviation and standard deviation for multiple species are also calculated in Appendix S1, as is the combined confidence interval.

The above analysis assumes that the correlation coefficients between individual covers (correlation *r*_*ij*_ between covers *i* and *j*) are negligible. If the correlation coefficients are not negligible, the covariance matrix needs to be created, and the following equation replaces Equation (11).

(12) $${\hat{d}}_{e}=\sqrt{\sum_{i=1}^{t}\sum_{j=1}^{t}r_{\mathrm{ij}}d_{i}d_{i}}$$

## DISCUSSION: GUIDELINES FOR RESEARCH DESIGN AND THE INTERPRETATION OF FIELD DATA

The point‐intercept method has long been popular among ecologists for measuring cover in the field; however, there are no firm rules for appropriate point‐intercept spacing or the number of point intercepts to sample. Even after data are collected, researchers do not have an established avenue for assessing error. We offer an explicit rule for point‐intercept spacing that is specific to the target species and study areas. We also developed formulas for the number of point intercepts that need to be sampled based on the level of accuracy that the researcher is seeking to attain for their data, and provide a user‐friendly spreadsheet (Appendix S1) that instantly calculates this quantity. After data collection, our spreadsheet calculates the accuracy of the final data set. Quadrat characteristics (e.g., point‐intercept spacing, number of point intercepts) can be easily calculated to establish a field protocol, and when the fieldwork is complete, the accuracy can (and should) be reported in ecological studies that use this technique.

### The spacing of point intercepts

All simulations showed that the accuracy of the estimated cover stabilizes at a point‐intercept spacing of at least 80% of the diameter of the widest plant; thus, if the widest individual plant is not expected to exceed 100 cm, for example, the point intercepts should be spaced at least 80 cm apart. If this spacing rule is not followed, the expected deviation increases considerably. For 40% cover and 100 point intercepts, for example, the expected deviation with >80% point‐intercept spacing is about 4%, but increases to about 14% when a point‐intercept spacing of 20% is applied (see Figure 2). The 80% rule is applicable for all numbers of point intercepts and cover values. Spacing can be increased when the size of the largest individual is uncertain as there is no statistical disadvantage to wider spacing, although the area sampled would be larger.

It is important to recognize that each species does not require its own quadrat. Because the error curve flattens for >80% spacing, any quadrat established for one species can be used for all species with smaller individuals (Pardo et al., 2013; Kent et al., 2018; Lévesque et al., 2018). Thus, a researcher may use one quadrat for the understory based on the largest individual of those species, with a second quadrat for the overstory or for larger species such as trees, or could even use one large quadrat with wide spacing to sample all species.

### Establishing the number of point intercepts for fieldwork

The number of point intercepts needed to yield a desired standard deviation, expected deviation, or confidence interval for any cover value (0–100%) can be computed using Appendix S1 and used to guide the field protocol choice. The largest number of point intercepts are needed when cover is 50%. If a species may have a cover of 50%, use Appendix S1 (Equations (7) and (10)), and either use that *n* only for those species that require more intercepts while decreasing the *n* for the others, or use the higher *n* for all species. If there is any uncertainty, err toward a 50% cover. If a researcher expects that the cover of every species sampled will be well below 50% (such as predictably low values in a desert), or well above 50% (e.g., a monospecific stand), the minimum number of point intercepts needed may be lower. Appendix S1 generally uses Equations (6) and (9) to calculate the expected deviation and confidence interval size, respectively. If the researcher believes that no species will have a cover of more than 20%, for example, *p* = 0.20 or $\frac{k}{n}=0.2$ could be used in the calculation (rather than a value between 0 and 0.20). If the researcher chooses to modify the number of intercepts from 50%, the confidence interval size or expected error may be larger than desired. A point‐intercept spacing of at least 80% is assumed.

The size of the confidence interval, the expected deviation, and the standard deviation are approximately inversely proportional to $\sqrt{n}$; thus, if the predetermined standard deviation, expected deviation, or the size of the confidence interval are cut by half, for example from 10% to 5%, the required number of point intercepts is approximately quadrupled.

### Accuracy of field data after data collection

#### Expected deviation and standard deviation of collected data

The expected deviation (*d*_*e*_) between the measured cover and the actual cover and the standard deviation can be calculated using Appendix S1 or Equation (3), which has been confirmed by simulation. If the 80% spacing rule is not followed, *d*_*e*_ will be larger than calculated.

The expected deviation and standard deviation are rather insensitive to the actual cover (*p*) when *p* is close to 0.50; for example, for *n* = 64: if *p* = 0.50, then *d*_*e*_ = 0.050 = 5.0%, while at *p* = 0.30, *d*_*e*_ only drops to 4.6%, and at *p* = 0.15 it is 3.6% (calculated in Appendix S1).

#### Confidence interval of the actual cover

The confidence interval can be calculated using Appendix S1 or with Equation (5). Each side of the confidence interval must be calculated separately as it is not symmetric around $\frac{k}{n}$ except for a 50% measured cover. The asymmetry of the confidence interval increases as the cover values get closer to 0% or to 100%. Because ecological data sets typically include rare or uncommon species, confidence interval asymmetry is expected in ecological data sets.

#### Combining multiple species

To calculate the confidence interval, standard deviation, or expected deviation for the total combined cover of two or more species, first find the estimates for individual species. The combined (measured) cover is the sum of individual measured covers. Appendix S1 and Equation (11) calculate the expected deviation, standard deviation, and the confidence interval of the total cover when correlations are negligible. If the correlation coefficients are not negligible, the covariance matrix needs to be found and Equation (12) used as described in the Methods (under “Combining multiple species”).

### Rare species

There may be relatively rare species in the study area that are not sampled by any point intercept, and therefore missed during field collection. A species with an actual cover of 1% is expected to cover 1 of 100 point intercepts; however, the probability that no point intercepts capture this species is 0.99^100^ = 37% calculated from the binomial distribution. For 400 point intercepts, the probability drops to 0.99^400^ = 1.8%. When a species exists at the study site but covers zero points (is unsampled), substituting *k* = 0 into Equation (5) gives a confidence interval of 0% to 3.7% for 95% confidence and *n* = 100. This means that there is only a 5% chance (95% confidence) that a species with cover exceeding 3.7% will not be sampled with 100 point intercepts. For a species with 1% cover, 384 point intercepts are needed to reach a 95% confidence that it will be sampled, while 663 point intercepts are needed for 99% confidence (calculated using Equation (10), or using Appendix S1). Increasing the total number of point intercepts greatly increases the probability of sampling infrequent species. If a researcher seeks to identify rare species with low cover values (e.g., less than 1%), they may opt to identify them when encountered and then assign a default cover value (e.g., 1%) (Drezner, 2003). Quantitative methods are of limited use for estimating the cover of trace species.

### Considerations, assumptions, and field preparation

Once the number of point intercepts is selected, sampling can be done using one large quadrat or combining multiple smaller quadrats. For example, larger vegetation may necessitate a quadrat with fewer point intercepts logistically, but that quadrat can be reused in several places in the study area to attain the total number of required point intercepts, as long as the 80% rule is maintained.

While we describe sampling through the use of quadrats throughout this paper, this term does not limit the scope of our results. Any randomized sampling that does not violate the point‐intercept distance rule can be used; for example, 10 transects with 20 point intercepts each (for 200 point intercepts) is acceptable under the current results presented here, as long as no two point intercepts are closer than the 80% minimum point‐intercept spacing on and across transects. Quadrats should be placed randomly, but can be placed in random directions or all oriented in the same direction. Also, quadrats do not have to be square in shape (e.g., Ji et al., 2019); they can be configured in any way that maintains the minimum distance between point intercepts.

Some important points and assumptions to keep in mind are as follows: (1) All guidelines are for one species in one canopy layer, except where specified otherwise. (2) We assume that each species has a cover value of 0–100%. (3) Multiple leaves on the same plant intersecting the same point would only be counted once, as would be the cover of two plants of the same species covering a shared space. Because each species is sampled individually, different species covering the same point‐intercept are counted separately. (4) If a species has over 100% cover due to being in two canopy layers, this will only be captured if the researcher distinguishes between the two (or more) layers during sampling, such as sampling overstory and understory vegetation separately. If this is done, combining the understory and overstory cover for the same species is done by treating these as two different species and calculating the total cover as described under “Combining multiple species.” (5) Calculations for multiple species (e.g., total cover) are also provided in Appendix S1.

It is important to recognize the limitations of the point‐intercept method, such as the seemingly large samples needed to attain reasonable levels of accuracy. A researcher need not be deterred from using the point‐intercept approach, however; while 400 point intercepts (for example) may seem high, a 10 × 10 quadrat (100 point intercepts) simply needs to be repeated four times to reach this sample size. Many researchers already intuitively sample repeatedly in the same area to strengthen results and accuracy. We now offer mathematically based tools that enable a researcher to establish field protocols for their target levels of accuracy and report the accuracy of their results. Our results also provide a mechanism for assessing past studies to ascertain their accuracy. There may also be a lingering perception that the point‐intercept technique is less reliable than other common techniques; however, this is not necessarily the case. Not only does this study provide an accurate assessment of the point‐intercept method, but comparisons with other cover‐assessment techniques cannot be made without similar efforts to assess the accuracy of the other techniques.

## AUTHOR CONTRIBUTIONS

T.D.D. developed the ideas, provided the ecological background, and developed the parameters for the simulations. Z.D. derived the formulas and performed the simulations. Both authors contributed to the writing and approved the final version of the manuscript.

## Supporting information


**Appendix S1.** Self‐calculating spreadsheet for the calculations developed in this paper.

## APPENDIX 1 SIMULATION PROGRAM IN FORTRAN

The evaluation is performed for each value of *n* (number of intercepts provided by the user) in three nested loops. Loop on 9% covers, nesting a loop on 20% intercept spacing, nesting a loop generating 100 study areas. Plants are generated in the study area until the percentage cover is achieved, and then tracts are generated for a total of 100,000 tracts for each percentage cover and intercept spacing.

## APPENDIX 2 SIMULATION DETAILS

### Establishing actual cover

We created a square study area with sides that are 200 times the average plant radius. We generated a random distribution of plants in the study area for a given actual cover (e.g., 10%). Because some plants centered outside the study area may cover some of the study area, the centers of plants are generated in a larger square that is wider in each direction by the maximum plant radius. The center and radius of each plant are randomly generated.

First, a grid of 2000 by 2000 points is established. All 4 million grid points begin with an assigned cover of 0, and a running count of covered points is initiated. When a plant is generated, the grid points (out of 4 million) that are under its canopy are identified as covered. Only points that were not already covered by previous plants (of the same species) are added to the total number of covered points. The number of points covered by the generated plants on a grid of 4 million points determine the actual cover; for example, if 400,000 points are covered, the actual cover is 10%. Plants are generated until the total number of covered points is within 0.01% of the specified actual cover for that simulation; for example, for a 10% actual cover, if the total number of covered points is less than 9.99%, more plants are added to the list. Once the accumulated cover exceeds 9.99%, no more plants are generated. If it exceeds 10.01%, the whole process restarts from no cover. For each of the nine cover values from 10% to 90%, 100 lists of plants that provide the required cover are generated.

### Establishing measured cover

Once a list of plants that provide the required cover is established, it is used to generate 1000 quadrats (representing field data) for each *n* and intercept spacing (10% to 200% of the largest plant diameter). The process is repeated 100 times for a total of 100,000 measurements for each of the 720 combinations (nine cover values, four numbers of intercepts, and 20 intercept‐spacing values). Quadrats are generated by randomly selecting the bottom left corner of the quadrat in the study area while confirming that the quadrat is inside the study area. Each intercept in the quadrat is checked against the list of plants to determine whether it is covered or not. The number of covered intercepts is established and compared with the actual cover to establish the expected deviation. The expected deviation for each of the 720 combinations is an average of 100,000 differences between the measured and actual cover.

### Accuracy of results

The purpose of any simulation study is to estimate the performance of a procedure to an acceptable degree of accuracy. Each of the 720 measured cover values is an average of 100,000 simulated results. We show that the average of 100,000 points is sufficient when the results are reported to one decimal point: the standard deviation of each result for *n* intercepts is $\sqrt{\frac{p(1-p)}{n}}$. Therefore, the standard deviation of the average, *σ*, is $\sqrt{\frac{p(1-p)}{100,000n}}$. For a 95% confidence interval, we use *z* = 1.96, so $=1.96\times \sqrt{\frac{p(1-p)}{100,000n}}$. Because *p*(1 – *p*) ≤ 0.25 for any *p*, $z\sigma =1.96\times \sqrt{\frac{p(1-p)}{100,000n}}\leq 1.96\times \sqrt{\frac{0.25}{100,000n}}=0.098\%\sqrt{\frac{10}{n}}$.

For example, the simulation result for the expected deviation for *p* = 20% and *n* = 225 intercepts is 2.1% (Table 2). For a 95% confidence level, $z\sigma =1.96\times \sqrt{\frac{0.2(1-0.2)}{100,000\times 225}}=0.017\%$; therefore, we can say with 95% confidence that the expected deviation is between 2.083% and 2.117%, which rounds to 2.1%. The *zσ* for the 95% confidence interval for any value of *p* and *n* = 225 cannot exceed $0.098\%\sqrt{\frac{10}{225}}=0.021\%.$
